# Effect of Denosumab or Alendronate on Vascular Calcification: Secondary Analysis of SALTIRE2 Randomized Controlled Trial

**DOI:** 10.1161/JAHA.123.032571

**Published:** 2024-09-09

**Authors:** Jolien Geers, Rong Bing, Tania A. Pawade, Mhairi K. Doris, Marwa Daghem, Alexander J. Fletcher, Audrey C. White, Laura Forsyth, Emily Evans, Jacek Kwieciński, Michelle C. Williams, Edwin J. R. van Beek, Soongu Kwak, Frederique E.C.M. Peeters, Evangelos Tzolos, Piotr J Slomka, Christophe Lucatelli, Stuart H. Ralston, Bernard Prendergast, David E. Newby, Marc R. Dweck

**Affiliations:** ^1^ BHF Centre for Cardiovascular Science University of Edinburgh Edinburgh UK; ^2^ Department of Cardiology Universitair Ziekenhuis Brussel (UZ Brussel) Vrije Universiteit Brussel (VUB) Brussels Belgium; ^3^ Department of Child Health University of Glasgow Glasgow UK; ^4^ Edinburgh Clinical Trials Unit University of Edinburgh Edinburgh UK; ^5^ Edinburgh Clinical Research Facility University of Edinburgh Edinburgh UK; ^6^ Department of Interventional Cardiology and Angiology Institute of Cardiology Warsaw Poland; ^7^ Edinburgh Imaging University of Edinburgh Edinburgh UK; ^8^ Department of Internal Medicine Seoul National University Hospital Seoul South Korea; ^9^ Maastricht University Medical Centre Maastricht The Netherlands; ^10^ Departments of Biomedical Sciences and Medicine Cedars‐Sinai Medical Center Biomedical Imaging Research Institute Los Angeles CA USA; ^11^ Institute of Genetics and Molecular Medicine University of Edinburgh UK; ^12^ St Thomas’ Hospital and Cleveland Clinic London London UK

**Keywords:** alendronate, computed tomography, denosumab, positron emission tomography, vascular calcification, Computerized Tomography (CT), Nuclear Cardiology and PET, Atherosclerosis

## Abstract

**Background:**

Patients with osteoporosis demonstrate increased vascular calcification but the effect of osteoporosis treatments on vascular calcification remains unclear. The present study aimed to examine whether coronary or aortic calcification are influenced by denosumab and alendronic acid treatment.

**Methods and Results:**

In a double‐blind randomized controlled SALTIRE2 (Study Investigating the Effect of Drugs Used to Treat Osteoporosis on the Progression of Calcific Aortic Stenosis) trial, patients with aortic stenosis were randomized 2:1:2:1 to denosumab, placebo injection, alendronic acid, or placebo capsule. Participants underwent serial imaging with computed tomography and 18F‐sodium fluoride positron emission tomography for the assessment of vascular calcium burden and calcification activity, respectively. We report the prespecified secondary analyses of 24‐month change in coronary calcium score, and 12‐month changes in thoracic aorta calcium score, coronary and aortic 18F‐sodium fluoride activity. One hundred fifty patients with aortic stenosis (72±8 years; 21% female) were randomized to denosumab (n=49), alendronic acid (n=51), and placebo (injection n=25, capsule n=25). There were no differences in change in coronary calcium scores between placebo (16 [−64 to 148] Agatston units) and either denosumab (94 [0–212] Agatston units, *P*=0.24) or alendronic acid (34 [−62 to 134], *P*=0.99). There were no differences in change in thoracic aorta calcium scores between placebo (132 [22–512] Agatston units) and either denosumab (118 [11–340], *P*=0.75) or alendronic acid (116 [26–498] Agatston units, *P*=0.62). There were no differences in changes in coronary or aortic 18F‐sodium fluoride activity between treatment groups.

**Conclusions:**

Neither alendronic acid nor denosumab are associated with changes in the activity or progression of coronary or aortic calcification. Osteoporosis treatments do not appear to have major impact on vascular calcification of atherosclerosis.

**registration:**

https://www.clinicaltrials.gov; Unique identifier: NCT02132026.

**Nonstandard Abbreviations and Acronyms**
18F‐NaF: 18F‐sodium fluoride
AU: Agatston units
CMA: coronary microcalcification activity
SALTIRE2: Study Investigating the Effect of Drugs Used to Treat Osteoporosis on the Progression of Calcific Aortic Stenosis

**Clinical Perspective**

**What Are the Clinical Implications?**

- Neither denosumab nor alendronic acid, 2 common treatments for osteoporosis that target processes of active calcification, cause amelioration or acceleration of coronary or aortic calcification.
- These drugs can be administered safely in patients with coexisting aortic stenosis or atherosclerosis.

**What Are the Clinical Implications?**

- Other pathways need to be explored to develop treatments capable of slowing calcification in the vasculature, which remains an important unmet clinical need.

There is considerable overlap in the pathophysiological processes driving skeletal bone formation and vascular calcification, including osteoblast activity, osteoclast activity, and the expression of osteoprotegerin, the receptor activator of NF‐κB ligand (RANKL) and NF‐κB (RANK).^1^, ^2^, ^3^ Many patients with atherosclerosis also have coexistent osteoporosis for which they will receive bisphosphonates or denosumab treatment.^4^, ^5^ Bisphosphonates prevent osteoclast‐mediated bone resorption by binding to hydroxyapatite.^6^ Denosumab is a monoclonal RANKL‐directed antibody, which prevents RANK receptor binding, thereby reducing osteoclast number and activity and resulting in decreased bone resorption.^7^ The pathological pathways targeted by these drugs are key processes implicated in both osteoporosis and atherosclerosis. There is, therefore, considerable interest in whether osteoporosis treatments might also have an impact on vascular calcification and atherosclerosis. Preclinical studies suggest that bisphosphonates and denosumab reduce the production of proinflammatory cytokines and inhibit arterial and valvular calcification.^8^, ^9^, ^10^ By contrast, observational data suggest that patients with osteoporosis on these treatments may demonstrate increased vascular calcification,^11^ although untangling the effects of the medication and the underlying condition for which they were prescribed is challenging. Indeed, bisphosphonates and denosumab are prescribed to patients with osteoporosis, a condition that itself is thought to increase the progression of vascular calcification. This key problem will always confound observational studies that seek to assess the isolated impact of these drugs on atherosclerosis. Ultimately, prospective randomized controlled trials are required to overcome confounding and determine whether osteoporosis treatments affect vascular calcification, and if so, whether this effect is stimulating or inhibiting.

The potential value of coronary artery calcium in identifying patients with coronary atherosclerosis was evident even with early studies using chest radiography or fluoroscopy.^12^ In current practice, modern computed tomography (CT) scans allow detailed noninvasive detection, localisation, and quantification of vascular calcification in both the aorta and coronary arteries, providing an important assessment of atherosclerotic plaque burden and powerful prognostic markers. CT calcium scoring provides assessment of the calcium burden in these territories and a surrogate of the overall atherosclerotic plaque burden that serves as a powerful prognostic marker across a wide range of patient populations.^13^ However, CT is unable to detect developing microcalcifications, and to distinguish between active and inactive disease. 18F‐sodium fluoride (18F‐NaF) is an established positron emission tomography (PET) tracer that detects novel areas of bone formation and remodeling,^14^ and that allows assessment of vascular calcification activity, and detection of developing calcium beyond the resolution of CT.^15^ Coronary 18F‐NaF uptake can be quantified across the coronary vasculature and is associated with more rapid coronary artery calcium score progression, high‐risk atherosclerotic plaque and future myocardial infarction.^16^, ^17^, ^18^, ^19^ Similarly, aortic 18F‐NaF PET can be quantified across the ascending aorta and aortic arch demonstrating associations with subsequent disease progression and the future risk of stroke.^20^

In a secondary analysis of a randomized controlled trial, we used these multi‐modality imaging approaches to investigate whether common osteoporosis treatments (alendronate and denosumab) influence vascular calcification in the aorta and coronary arteries.

## Methods

### Trial Design and Protocol

The SALTIRE2 (Study Investigating the Effect of Drugs Used to Treat Osteoporosis on the Progression of Calcific Aortic Stenosis) trial was a single‐center parallel group double‐blind randomized controlled trial with Trial Steering Committee oversight and regional ethics committee (Scotland A Research Ethics Committee, 14/SS/0064) and Medicines and Healthcare products Regulatory Agency (EudraCT 2014–001112‐19) approvals. The study was conducted in accordance with the Declaration of Helsinki and registered on ClinicalTrials.gov (NCT02132026). All patients provided written informed consent. The data that support the findings of this study are available from the corresponding author upon reasonable request.

The trial population and full protocol have been published previously.^21^ In brief, patients over 50 years of age with a peak aortic jet velocity>2.5 m/s on Doppler echocardiography and grade 2–4 aortic valve calcification on semiquantitative echocardiographic assessment were recruited from cardiology outpatient clinics. Major exclusion criteria included anticipated or planned aortic valve surgery in the next 6 months, life expectancy <2 years, treatment for osteoporosis with bisphosphonates or denosumab, and long‐term corticosteroid use. Participants underwent comprehensive baseline assessments including echocardiography, combined 18F‐NaF PET‐CT and non‐contrast coronary CT calcium scans. Participants were randomized in a 2:1:2:1 ratio using computer‐based methods to either subcutaneous denosumab (Prolia, Amgen, CA) 60 mg every 6 months, placebo injection every 6 months, oral alendronic acid (TEVA UK, UK) 70‐mg once weekly or matching placebo capsule once weekly. A minimization algorithm with a random component was used, incorporating age (<73 and ≥73 years), sex, presence or absence of a bicuspid valve and baseline aortic valve calcium scores (≤1607 and >1607 Agatston units [AU]). Follow‐up visits were performed at 6, 12, 18, and 24 months. Repeat 18F‐NaF PET‐CT and non‐contrast CT were performed at 12 months and a further non‐contrast CT performed at 24 months. Where possible, participants who were subsequently scheduled for aortic valve replacement had their pending 12‐ or 24‐month visit brought forward, after which the trial intervention was discontinued.^21^

### Imaging

A full description of image acquisition has been published previously.^21^ In brief, non‐contrast coronary CT calcium scoring (120 kV CARE Dose4D [Siemens], 3‐mm slice thickness, spiral acquisition, 70% R‐R interval, inspiratory breath‐hold) and 18F‐NaF contrast‐enhanced PET‐CT were performed on a 128‐multislice scanner (Biograph mCT, Siemens, Germany) in a dedicated research imaging center (Edinburgh Imaging, University of Edinburgh) at baseline and at 12 months using prospective ECG gating and standardized protocols. PET image acquisition was performed ~60 minutes after intravenous injection of 125 MBq 18F‐NaF with a single bed position centered on the heart. A further CT coronary calcium scan was repeated at 24 months.

### Image Analysis

Coronary artery calcium scores were semi‐automatically calculated using Vitrea v6.9.68.1 (Vitrea Advanced, Vital Images, Minnetonka, USA) and standard weightings, with analysis only undertaken in those patients without stents or prior coronary artery bypass grafts. Thoracic aorta calcium scores were calculated across the ascending aorta, aortic arch and descending aorta using the same software on attenuation correction CT scans using OsiriX version 12.0.0 (Bernex), as described previously.^22^ Briefly, calcium scores in the different segments of the thoracic aorta were calculated as follows: (A) measurements for the ascending aorta started from the sinotubular junction and ended 1 slice below the aortic arch, (B) the aortic arch ranged from the most inferior slice at which the ascending and descending aorta were contiguous to the origin of the great vessels, and (C) the descending aorta was scored from just distal to the left subclavian artery to the proximal aspect of the coeliac axis.

Coronary artery 18F‐NaF uptake was quantified with FusionQuant v1.20.05.14 software (Cedars‐Sinai, CA, USA), providing an assessment of coronary microcalcification activity (CMA) across all 3 coronary arteries.^23^ In brief, 3‐dimensional volumes of interest created around a centerline drawn from the proximal to distal segments of each of the 3 main epicardial arteries were extracted, the left main coronary artery being included with the left anterior descending artery. CMA was then quantified on fused and co‐registered PET‐CT images as the total standardized uptake value (SUV) units in each volume of interest above a set threshold of mean blood pool SUV (right atrium) plus 2 SD.

Aortic 18F‐NaF uptake was quantified in the ascending aorta and arch using 2 different image analysis techniques with FusionQuant v1.20.05.14 software (Cedars‐Sinai, CA, USA): the descending aorta was not assessed because of overspill of 18F‐NaF signal from the adjacent vertebra. First, we measured the aortic microcalcification activity, as described previously.^24^ In brief, using non‐contrast CT and 18F‐NaF PET images, a centerline extending from the sinotubular junction to the point immediately distal to the left subclavian artery was drawn with a diameter set to the maximal luminal diameter of the aorta +4 mm, accounting for the spatial resolution of PET imaging. The aortic activity was calculated by dividing the cumulative voxel intensity in this region of interest by the volume in cm^3^, to give aortic intensity per cm^3^. The background radiotracer activity was measured in a similar way, namely by dividing the cumulative radiotracer activity in two 2‐cm^3^ atrial volumes of interest (in right and left atrium respectively) by the volume, providing a background voxel intensity per cm^3^. The aortic microcalcification activity represents the ratio of aortic activity to background radiotracer activity (Figure S1).

Second, we used the ‘most diseased segment’ method, as described previously.^25^ Serial polyhedrons 3 mm in height were drawn in the ascending aorta on axial slices of fused and co‐registered PET‐CT images, and the maximum and mean target‐to‐background ratio (TBR_max_, SUV_max_ divided by blood pool SUV_mean_; TBR_mean_, SUV_mean_ divided by blood pool SUV_mean_) calculated per slice. The most diseased segment TBR_max_ was taken as the mean of the 3 adjacent segments with the highest TBR_max_ measurements. The most diseased segment TBR_mean_ was calculated in the same manner.

### Trial End Points

The primary end point of the SALTIRE 2 trial was change in the aortic valve calcium score, which has been reported previously alongside other markers of aortic stenosis progression.^21^ Briefly, the original trial concluded that denosumab and alendronic acid have no effect on the progression of aortic valve calcification or stenosis severity over 24 months in patients with aortic stenosis. We here report the pre‐specified secondary end points of the calculated 24‐month change in coronary calcium score, 12‐month change in aortic calcium score as well as 12‐month change in coronary and aortic 18F‐NaF uptake.

Consistent with our approach for the primary end point of the trial [16], a daily rate of change was calculated, then multiplied by either 365 or 730 to provide the 12‐month and 24‐month change, respectively. That is: [(final visit measurement – baseline visit measurement) / days from baseline visit to final visit] * either 365 or 730. For those end points specified as 24‐month change, the 12‐month visit was used as the final visit if a participant did not attend a 24‐month visit.

### Statistical Analysis

Intention to treat analysis was undertaken. Both placebo groups were combined for the analysis. Categorical variables are presented as number (%) while continuous variables are presented as median (interquartile range) or mean±SD. Distributions of data were tested for normality with the Shapiro–Wilk test and quantile‐quantile plots. Between‐group differences were compared with the Wilcoxon rank sum test or Kruskal‐Wallis test, as appropriate. Sensitivity analysis for the change in coronary calcium score was performed after excluding scans with motion artifact. Where the baseline scan scores were inaccurate because of motion artifact, the scores from the 12‐month and 24‐month scans were used to calculate the 2‐year change if appropriate. Where the final scan scores were inaccurate because of motion artifact, the scores from the baseline scan and 12‐month scans were used to calculate the 2‐year change if appropriate. To consider repeated measurements, mixed‐effects linear regression models were constructed for each treatment arm. This was done fitting a mixed model with: treatment, time point, and baseline as fixed effects and participant as a random effect. Least square means for each active trial arm model were calculated and compared with placebo separately. Analysis was performed using SAS Enterprise Guide v 7.15 (SAS Institute Inc., Cary, NC, USA). A 2‐sided *P* value of <0.05 was considered statistically significant.

## Results

Between August 2015 and November 2017, 199 patients were consented, of whom 150 (49 denosumab, 51 alendronic acid, 50 placebo) were included in the trial, as described previously.^21^ Baseline characteristics were balanced between study arms (Table 1). The mean age was 73±8 years and 21% of the cohort were female. There was a high prevalence of hypertension (76%) and hypercholesterolemia (61%), and more than half of participants were prior or current smokers. A history of myocardial infarction, percutaneous coronary intervention or coronary artery bypass grafting was present in 11%, 22%, and 10%, respectively. The prevalence of prior transient ischaemic attack or stroke was 13%.

**Table 1:** Baseline Participant Characteristics

Characteristic	Overall (n=150)	Placebo (n=50)	Denosumab (n=49)	Alendronate (n=51)
Age, y	73±8	72±7	72±8	73±8
Women	31 (21%)	10 (20%)	11 (22%)	10 (20%)
White Scottish	136 (91%)	46 (92%)	44 (90%)	46 (90%)
Hypertension	114 (76%)	41 (82%)	35 (71%)	38 (75%)
Hypercholesterolemia	91 (61%)	35 (70%)	34 (69%)	22 (43%)
Type 2 diabetes	35 (23%)	12 (24%)	12 (24%)	11 (22%)
Chronic kidney disease	12 (8%)	2 (4%)	6 (12%)	4 (8%)
Chronic liver disease	1 (0.7%)	1 (2.0%)	0 (0%)	0 (0%)
Osteoporosis	0 (0%)	0 (0%)	0 (0%)	0 (0%)
Prior angina	39 (26%)	13 (26%)	12 (24%)	14 (27%)
Previous myocardial infarction	17 (11%)	4 (8%)	8 (16%)	5 (10%)
Previous percutaneous coronary intervention	33 (22%)	11 (22%)	9 (18%)	13 (25%)
Previous coronary artery bypass graft surgery	15 (10%)	8 (16%)	3 (6%)	4 (8%)
Previous transient ischemic attack/cerebrovascular accident	20 (13%)	6 (12%)	9 (18%)	5 (10%)
Malignancy	31 (21%)	10 (20%)	8 (16%)	13 (25%)
Current smoker	13 (9%)	3 (6%)	2 (4%)	8 (16%)
Ex‐smoker	77 (51%)	27 (54%)	26 (53%)	24 (47%)
Height, m	1.71±0.09	1.70±0.08	1.71±0.10	1.71±0.08
Weight, kg	86 [76 to 93]	85 [79 to 91]	85 [76 to 91]	88 [76 to 100]
Systolic blood pressure, mm Hg	150±19	150±19	149±20	150±20
Diastolic blood pressure, mm Hg	78±11	77±10	79±12	76±11
Heart rate, beats per min	67 [59 to 77]	70 [62 to 76]	67 [59 to 80]	66 [56 to 75]
C‐terminal telopeptide, μg/L	0.22 [0.16 to 0.30]	0.22 [0.17 to 0.30]	0.23 [0.18 to 0.32]	0.20 [0.14 to 0.27]

Values are median [interquartile range], mean±SD, or n (%).

As reported in the primary paper, baseline C‐terminal telopeptide concentrations were similar between treatment groups (Table 1) and halved from baseline to 6 months with both denosumab and alendronic acid but were unchanged with placebo (Table S1).

### Coronary Artery Calcification

**Figure 1:**
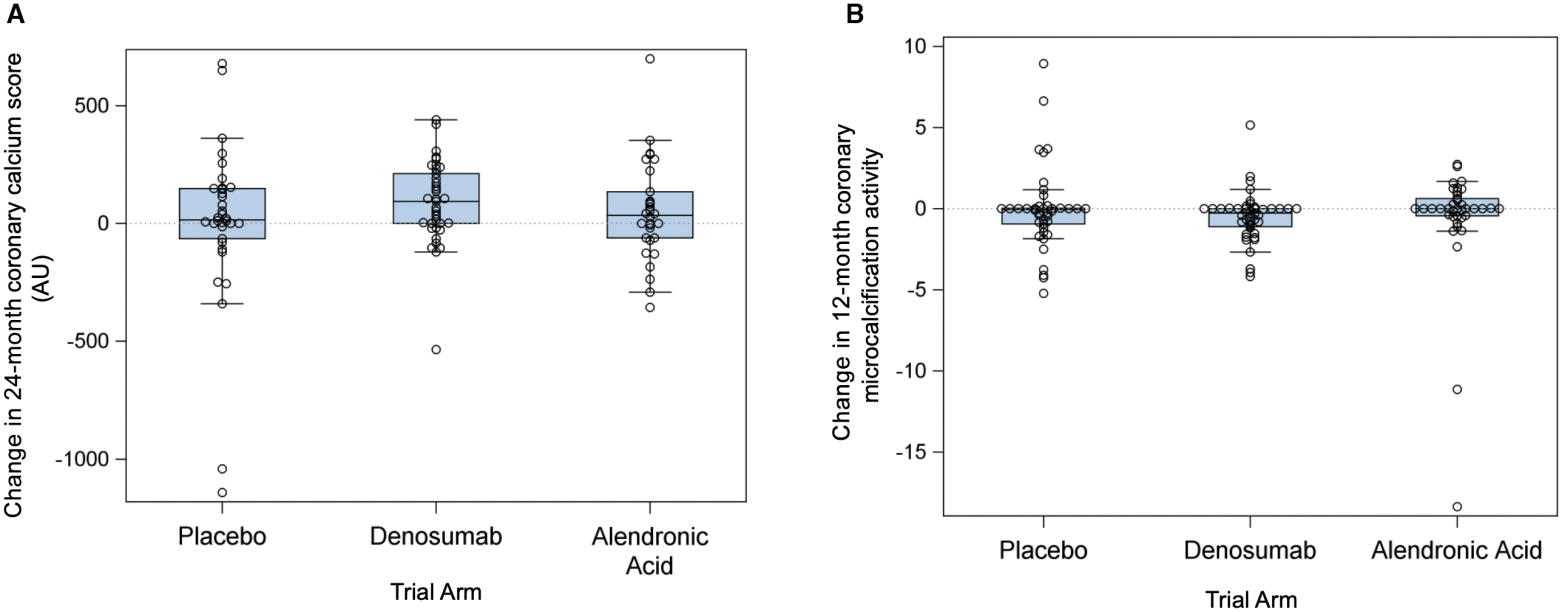
Change in coronary artery calcium score and 18F‐sodium fluoride uptake. **A**, Calculated change in 24‐month coronary artery calcium score (*P*=0.24 for denosumab vs placebo; *P*=0.99 for alendronic acid vs placebo). **B**, Calculated change in 12‐month coronary artery microcalcification activity (*P*=0.47 for denosumab vs placebo; *P*=0.28 for alendronic acid vs placebo). AU indicates Agatston units.

Twenty‐four‐month change in coronary artery calcium score was calculated in 85 participants (29 placebo, 32 denosumab, 24 alendronic acid). The overall median baseline coronary artery calcium score was 327 (46 to 998) Agatston units (AU) and the overall median 24‐month change was 39 (−18 to 151) AU. There were no statistically significant differences in 24‐month change between denosumab and placebo (94 [0 to 212] versus 16 [−64 to 148] AU, *P*=0.24) or between alendronic acid and placebo (34 [−62 to 134] versus 16 [−64 to 148] AU, *P*=0.99) (Figure 1A). Sensitivity analysis excluding coronary calcium scores affected by artifact in 6 patients also demonstrated no statistically significant differences in 24‐month change between denosumab and placebo (67 [0 to 191] versus 15 [−7 to 137] AU, *P*=0.20) or between alendronic acid and placebo (0 [−76 to 91] versus 15 [−7 to 137] AU, *P*=0.66).

Twelve‐month change in coronary artery 18F‐NaF uptake as assessed by CMA was calculated in 124 participants (42 placebo, 44 denosumab, 38 alendronic acid). The overall median baseline CMA score was 1.08 [0.00 to 2.69] and the overall median 12‐month change was −0.01 [−0.85 to 0.06]. There were no statistically significant differences in 12‐month change in CMA between denosumab and placebo (−0.27 [−1.12 to 0.00] versus −0.05 [−0.93 to 0.03], *P*=0.47) or between alendronic acid and placebo (0.00 [−0.44 to 0.62] versus −0.05 [−0.93 to 0.03], *P*=0.28) (Figure 1B).

### Aortic Calcification

**Figure 2:**
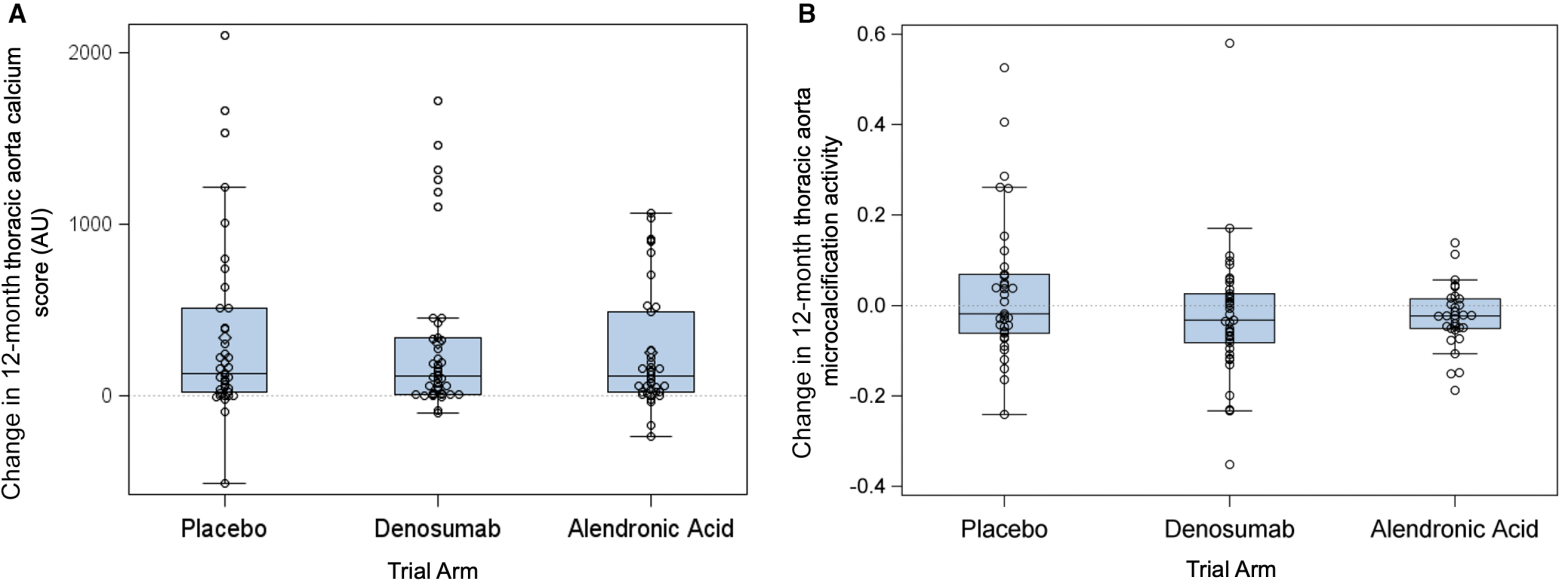
Change in aortic calcium score and 18F‐sodium fluoride uptake. **A**, Calculated change in 12‐month total thoracic aorta calcium score (*P*=0.75 for denosumab vs placebo; *P*=0.62 for alendronic acid vs placebo). **B**, Calculated change in 12‐month total aortic microcalcification activity (*P*=0.18 for denosumab vs placebo; *P*=0.24 for alendronic acid vs placebo). AU indicates Agatston units.

Twelve‐month change in thoracic aorta calcium score was calculated in 117 participants (39 placebo, 40 denosumab, 38 alendronic acid). The overall median baseline calcium score was 1074 (224 to 2429) AU in the thoracic aorta; 0 (0 to 6) AU in the ascending aorta; 671 (148 to 1458) AU in the aortic arch; and 229 (8 to 949) AU in the descending aorta. The overall median 12‐month change in calcium score was 127 (21 to 414) AU across the entire thoracic aorta; 0 (0 to 0) AU in the ascending aorta; 90 (13 to 221) AU in the aortic arch; and 24 (0 to 150) AU in the descending aorta. There were no statistically significant differences in 12‐month change in total thoracic aorta calcium score between denosumab and placebo (118 [11 to 340] versus 132 [22 to 512] AU, *P*=0.75) nor between alendronic acid and placebo (116 [26 to 498] versus 132 [22 to 512] AU, *P*=0.62) (Figure 2A, Table 2). Similarly, there was no statistically significant difference in the change in calcium score between treatment groups in the different regions of the thoracic aorta (Table 2).

**Table 2:** Twelve‐Month Change in Calcium Score Between Treatment Groups in the Different Regions of the Thoracic Aorta

Variable	Calcium score (AU)			
	Thoracic aorta	Ascending aorta	Aortic arch	Descending aorta
Overall	127 [21 to 414]	0 [0 to 0]	90 [13 to 221]	24 [0 to 150]
Placebo	132 [22 to 512]	0 [0 to 0]	96 [14 to 283]	16 [0 to 126]
Denosumab	118 [11 to 340]	0 [0 to 2]	83 [9 to 191]	21 [0 to 154]
Alendronic Acid	116 [26 to 498]	0 [0 to 0]	83 [11 to 283]	0 [29 to 156]

Values are median [interquartile range]. AU indicates Agatston units.

Twelve‐month change in aortic 18F‐NaF uptake as assessed by aortic microcalcification activity was calculated in 111 participants (38 placebo, 40 denosumab, 33 alendronic acid). The overall median baseline total aortic microcalcification activity was 1.07 (1.02 to 1.14); ascending aortic microcalcification activity was 1.05 (1.00 to 1.12); aortic arch microcalcification activity was 1.12 (1.04 to 1.22). The overall median 12‐month change in total aortic microcalcification activity was −0.02 (−0.07 to 0.04); ascending aortic microcalcification activity was −0.01 (−0.05 to 0.04); aortic arch microcalcification activity was −0.04 (−0.12 to 0.03). There were no statistically significant differences in 12‐month change in total aortic microcalcification activity between denosumab and placebo (−0.03 [−0.09 to 0.03] versus −0.02 [−0.06 to 0.07], *P*=0.18) or between alendronic acid and placebo (−0.02 [−0.05 to 0.01] versus −0.02 [−0.06 to 0.07], *P*=0.24) (Figure 2B). Similarly, using the most diseased segment approach, there were no statistically significant differences in the 12‐month change in aortic 18F‐NaF uptake between the treatment groups and placebo (Table S2).

## Discussion

**Figure 3:**
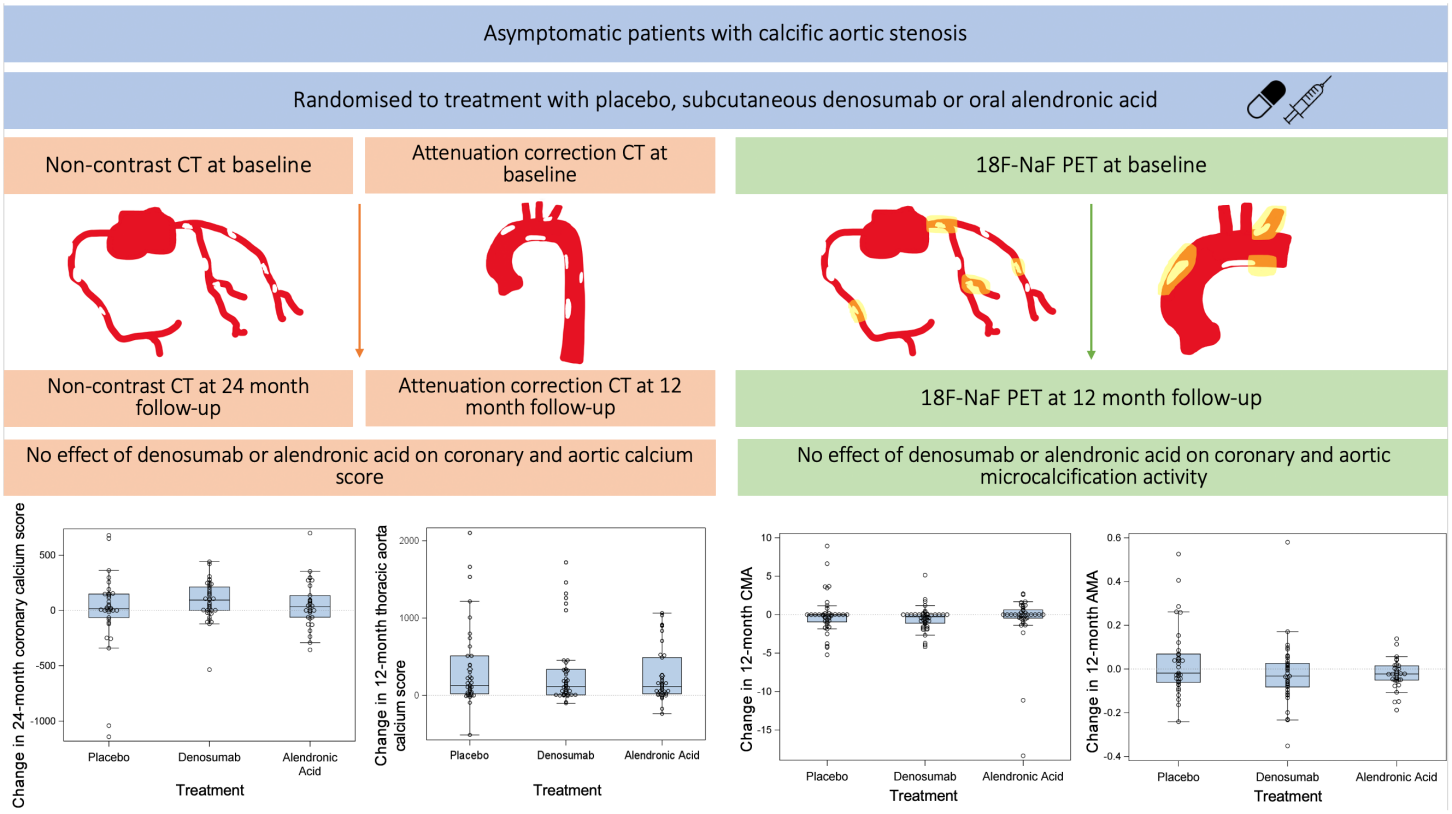
No effect of treatment with denosumab or alendronic acid on either aortic or coronary calcification in asymptomatic patients with aortic stenosis. Asymptomatic patients with calcific aortic stenosis were randomized to treatment with placebo, denosumab or alendronic acid. Vascular calcification in the coronary arteries was monitored by CT calcium scoring at baseline and 24‐month follow‐up; vascular calcification in the thoracic aorta was measured on attenuation correction CT scans at baseline and 12‐month follow‐up; coronary and aortic microcalcification activity were monitored by 18F‐NaF PET at baseline and 12‐month follow‐up. There were no effects of either drug on calcium scores or microcalcification activity in either the aorta or coronary arteries, indicating no effect on either the burden of calcific atherosclerosis or vascular calcification activity. 18F‐NaF PET indicates 18F‐sodium fluoride positron emission tomography; AMA, aortic microcalcification activity; CMA, coronary microcalcification activity; and CT: computed tomography.

In this single‐center parallel‐group double‐blind randomized controlled trial, we found that treatment with denosumab or alendronic acid had no effect on aortic or coronary calcification in patients with asymptomatic calcific aortic stenosis (Figure 3). This is consistent with the study's primary findings showing no demonstrable effect of these drugs on the progression of aortic valve calcification.^21^ We conclude that these anti‐osteoporotic drugs are not accompanied by changes in coronary or aortic calcification in patients with cardiovascular disease suggesting a neutral effect on overall vascular calcification.

There is considerable overlap in the pathophysiology of skeletal bone formation and vascular calcification with increased vascular calcification observed in patients with deficiencies in bone regulation, such as osteoporosis and Paget's disease.^2^, ^3^, ^26^ Both skeletal bone formation and vascular calcification are characterized by osteoblast and osteoclast activity, and the increased activity of bone morphogenic protein and the osteoprotegerin, receptor activator of NF‐κB (RANK) and receptor activator of NF‐κB ligand (osteoprotegerin/RANK/RANKL) pathway. Osteoprotegerin‐deficient mice develop both premature and severe osteoporosis as well as arterial calcifications,^27^ whilst increased serum osteoprotegerin concentrations in humans are associated with adverse cardiovascular outcomes.^28^, ^29^, ^30^, ^31^, ^32^ There has therefore been concern that drugs targeting bone metabolism (and the osteoprotegerin/RANK/RANKL pathway in particular) might also impact vascular calcification. This is of particular relevance given the association between vascular calcification and adverse cardiovascular events, and given that drugs targeting bone metabolism (such as bisphosphonates and denosumab) are widely prescribed for the treatment of osteoporosis in elderly patients with co‐existent atherosclerosis. Disentangling the effects of these drugs on vascular calcification is difficult in observational studies where there is the confounding effect of the underlying osteoporosis for which these drugs were prescribed.

In the SALTIRE 2 randomized controlled trial, we used state of the art cardiovascular imaging to assess the effects of alendronate and denosumab on both aortic valve and vascular calcification in patients with aortic stenosis. As mentioned in the primary paper, a 50% reduction of serum C‐terminal telopeptide concentration in patients receiving denosumab or alendronic acid could be observed, reflecting the pharmacodynamic effect of these drugs on bone turnover and resorption, and thereby demonstrating that the treatment was effective. Opposed to this, we observed no effect of either drug on calcium scores or 18F‐NaF PET activity in either the aorta or coronary arteries, indicating no effect on either the burden of calcific atherosclerosis or vascular calcification activity. These results were consistent regardless of the measure of calcification used, further bolstering confidence in the outcome. Our results are also similar to other less sophisticated randomized trial data of bisphosphonates and denosumab which reported no differences between treatment and placebo groups in progression of aortic calcification assessed on plain x‐rays.^33^, ^34^, ^35^ Whilst there appears to be overlap between the pathophysiology of skeletal bone formation and vascular calcification, modulating bone metabolism with drugs, such as alendronate or denosumab, does not appear to have any harmful or beneficial effects on vascular calcification. This is reassuring for patients receiving treatment for osteoporosis, although the development of treatments capable of slowing calcification in the vasculature and heart valves remains an important clinical priority and unmet need.

We recognize several limitations with our analysis. As acknowledged in the primary manuscript, the trial was conducted in a single center and composed of a largely White male population with normal bone health, which may affect generalizability of our results. Not all patients completed 2 years of follow‐up, largely because of aortic valve replacement occurring before trial completion. Vascular calcification is a slow process and we cannot rule out a potential difference between treatment and placebo groups occurring in the longer term as a consequence of the relatively short follow‐up period. Finally, the end points were imaging parameters only and the trial was not designed or powered to investigate clinical events, although both CT calcium scoring and 18F‐NaF PET have been closely associated with such clinical events in previous studies.^13^, ^15^, ^16^, ^17^, ^18^, ^19^, ^20^

In conclusion, in a randomized controlled trial of patients with asymptomatic aortic stenosis, neither alendronic acid nor denosumab were associated with a change in activity or progression of calcification within the coronary arteries or aorta, suggesting that these drugs neither promote nor inhibit vascular calcification.

## Sources of Funding

This trial was funded by the British Heart Foundation (FS/14/78/31020). MRD is supported by the British Heart Foundation (FS/SCRF/21/32010) and is the recipient of the Sir Jules Thorn Award for Biomedical Research 2015 (15/JTA). DEN (CH/09/002, RG/F/22/110093, RE/18/5/34216), MCW (FS/ICRF/20/26002) are supported by the British Heart Foundation. PDA is supported by a Heart Foundation of New Zealand Senior Fellowship (1844). EJRVB is support by the Scottish Imaging Network (www.sinapse.ac.uk). DEN is also the recipient of a Wellcome Trust Senior Investigator Award (WT103782AIA).

## Disclosures

MCW has given talks for Canon Medical Systems, Siemens Healthineers and Novartis, but with no conflict of interest for this paper. The remaining authors have no disclosures to report.

## Supporting information

Tables S1–S2
Figure S1
