# Supplementary material for: Effect of Denosumab or Alendronate on Vascular Calcification: Secondary Analysis of SALTIRE2 Randomized Controlled Trial
Source: J Am Heart Assoc. 2024 Sep 9;13(18):e032571. doi: 10.1161/JAHA.123.032571 (PMC11935633; doi:10.1161/JAHA.123.032571)
Supplement: Supplementary file 1 — Tables S1–S2 Figure S1 [file JAH3-13-e032571-s001.pdf]

SUPPLEMENTAL MATERIAL

Table S1. Baseline, 6-month follow-up and 6-month change in C-terminal telopeptide concentration between treatment groups.

Variable	C-terminal telopeptide (µg/L)		
	**Baseline**	**6-month follow-up**	**6-month change**
**Overall**	0.22 [0.16 to 0.30]	0.12 [0.08 to 0.20]	-0.06 [-0.11 to -0.01]
**Placebo**	0.22 [0.17 to 0.30]	0.26 [0.16 to 0.31]	0.00 [-0.04 to 0.05]
**Denosumab**	0.23 [0.18 to 0.32]	0.11 [0.08 to 0.17]	-0.09 [-0.15 to -0.06]
**Alendronic Acid**	0.20 [0.14 to 0.27]	0.09 [0.08 to 0.13]	-0.07 [-0.14 to -0.04]

Values are median [interquartile range].

Wilcoxon rank sum test for the change in C-terminal telopeptide from baseline to 6 months comparing treatment groups: $p<0.001$ for denosumab compared to placebo, $p<0.001$ for alendronic acid compared to placebo.

Table S2. Ascending aorta 18F-sodium fluoride uptake assessed by most diseased segment method.

	TBR_{mean}	TBR_{max}	SUV_{mean}	SUV_{max}
**Overall: baseline**	1.05 [0.97 to 1.17]	1.73 [1.53 to 1.90]	1.23 [1.07 to 1.42]	1.92 [1.63 to 2.32]
**Overall: 12-month change**	0.02 [-0.07 to 0.11]	0.02 [-0.07 to 0.11]	0.04 [-0.11 to 0.13]	0.05 [-0.12 to 0.27]
**Placebo: 12-month change**	0.03 [-0.06 to 0.09]	0.03 [-0.06 to 0.09]	0.00 [-0.15 to 0.12]	0.02 [-0.17 to 0.26]
**Denosumab: 12-month change**	0.02 [-0.05 to 0.13]	0.02 [-0.05 to 0.13]	0.05 [-0.10 to 0.11]	0.12 [-0.10 to 0.25]
**Alendronic Acid: 12-month change**	0.01 [-0.11 to 0.11]	0.01 [-0.11 to 0.11]	0.06 [-0.05 to 0.13]	0.05 [-0.10 to 0.29]

Values are median [interquartile range].

Wilcoxon rank-sum test for the change in ascending aorta 18F-NaF uptake as defined by TBR_{mean} from baseline to 12 months comparing treatment groups: p=0.54 for denosumab compared to placebo, p=0.84 for alendronic acid compared to placebo.

Figure S1. Method of measuring aortic microcalcification activity using FusionQuant software.

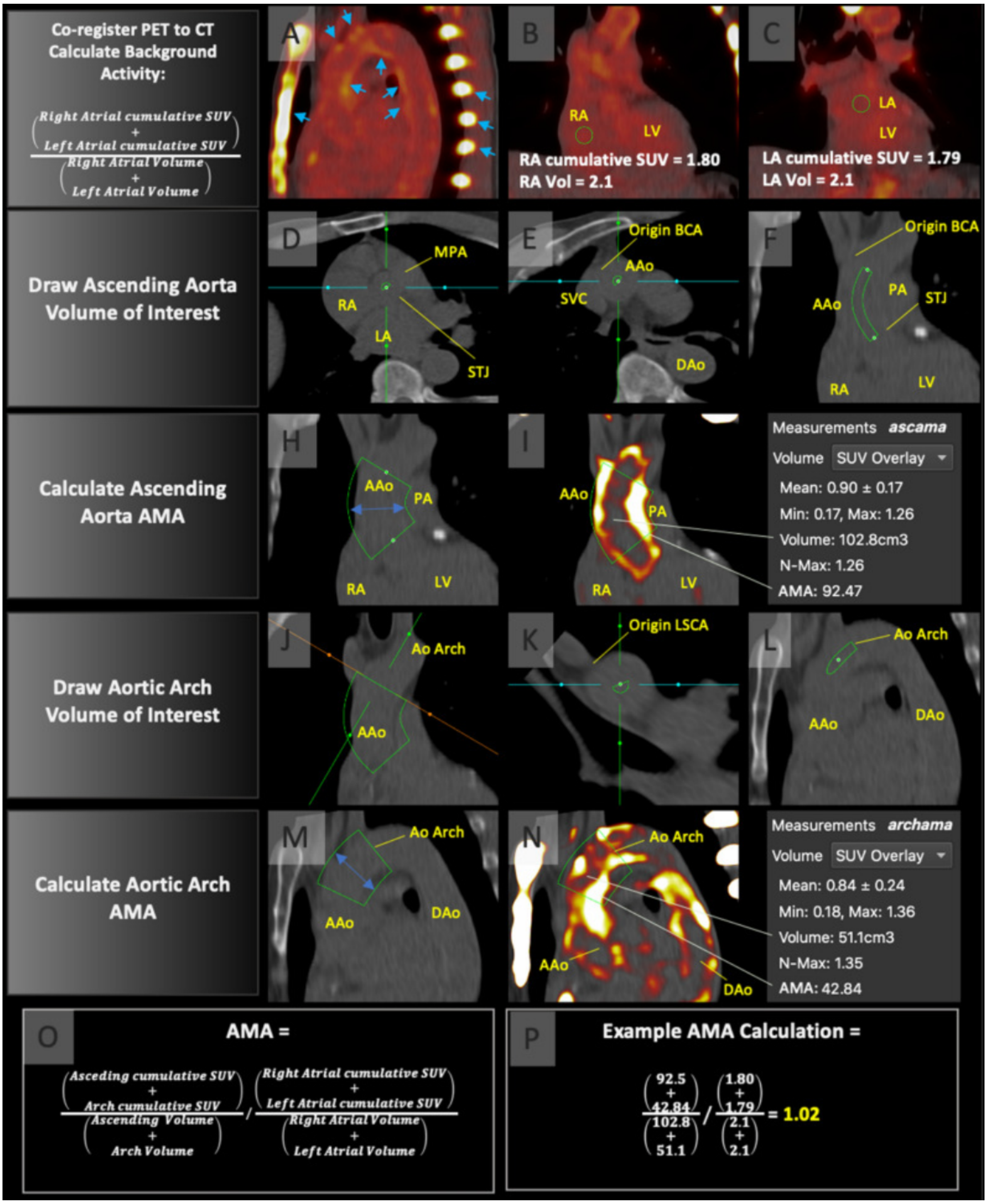

(**A**) Co-registration of PET signal in 3 orthogonal planes to non-contrast CT images using landmarks of sternum, spine, and aortic wall (blue arrows). (**B** + **C**) Background activity in the blood pool is determined as the average standardised uptake value (SUVmean) of two 2 cm^3 region of interest in the right (**B**) and left (**C**) atrium. (**D** + **E** + **F**) With the ^{18}F-sodium fluoride overlay turned off, a volume of interest around the ascending aorta is created using a centerline function in multiplanar reconstruction images. Perpendicular to the aorta, the volume of interest starts at the sinotubular junction (**D**) and finishes at the slice just proximal to the origin of the brachiocephalic artery (**E**). (**H**) The width of the volume of interest is increased to the maximum ascending aortic diameter + 4 mm. (**I**) The ^{18}F-sodium fluoride overlay is reinstated to ensure good coverage, and the ascending aortic AMA and volume are calculated. (**J** + **K** + **L**) Similarly, the aortic arch volume of interest is drawn, starting with the slice immediately distal to the ascending aortic volume of interest (**J**), and finishing with the slice after the origin of the left subclavian artery (**K**). (**M**) The width of the aortic arch volume of interest is increased to the maximal arch dimeter + 4 mm (**N**). The ^{18}F-sodium fluoride overlay is reinstated to check good coverage and calculate the aortic arch AMA and volume. (**O** + **P**) The following formula is used for calculating the overall AMA. Reprinted with permission from Fletcher et al. [20]. PET: positron emission tomography; CT: computed tomography; SUV: standard uptake value; AMA: aortic microcalcification activity; RA: right atrium; LA: left atrium; LV: left ventricle; MPA: mean pulmonary artery; STJ: sinotubular junction; BCA: brachiocephalic artery; SVC: superior vena cava; AAo: ascending aorta; DAo: descending aorta; PU: pulmonary artery; Ao Arch: aortic arch; LSCA: left subclavian artery.
